# An Efficient Adaptive Window Size Selection Method for Improving Spectrogram Visualization

**DOI:** 10.1155/2016/6172453

**Published:** 2016-08-24

**Authors:** Shibli Nisar, Omar Usman Khan, Muhammad Tariq

**Affiliations:** ^1^National University of Computer and Emerging Sciences, Peshawar 25000, Pakistan; ^2^Princeton University, New Jersey, NJ 08544, USA

## Abstract

Short Time Fourier Transform (STFT) is an important technique for the time-frequency analysis of a time varying signal. The basic approach behind it involves the application of a Fast Fourier Transform (FFT) to a signal multiplied with an appropriate window function with fixed resolution. The selection of an appropriate window size is difficult when no background information about the input signal is known. In this paper, a novel empirical model is proposed that adaptively adjusts the window size for a narrow band-signal using spectrum sensing technique. For wide-band signals, where a fixed time-frequency resolution is undesirable, the approach adapts the constant Q transform (CQT). Unlike the STFT, the CQT provides a varying time-frequency resolution. This results in a high spectral resolution at low frequencies and high temporal resolution at high frequencies. In this paper, a simple but effective switching framework is provided between both STFT and CQT. The proposed method also allows for the dynamic construction of a filter bank according to user-defined parameters. This helps in reducing redundant entries in the filter bank. Results obtained from the proposed method not only improve the spectrogram visualization but also reduce the computation cost and achieves 87.71% of the appropriate window length selection.

## 1. Introduction

Time-frequency analysis is typically required to characterize nonstationary phenomena such as speech [1, 2], biomedicine [3, 4], vibration [5], and music [6] based signals. The frequency contents for the analysis can be revealed if a Fourier transform is applied to these signals [7]. However, in doing so, all time related information will be lost [8]. The deficiency was first addressed in [9] where the Fourier transform was applied to analyze small sections of a signal at a time. Over time, this technique became popularly known as the Short Time Fourier Transform (STFT) [10, 11]. A significant shortcoming of the STFT is that it considers a fixed time-frequency resolution for all types of signals [12, 13]. This approach is not desirable for wide-band or ultrawide-band signals where low spectrogram resolutions can be observed. Moreover, the selection of an appropriate window size is vital for the STFT [14]. The window size should ideally ensure that the input signal falling within it should remain stationary [15]. However, if the window is too small, then the frequency domain cannot be localized [16].

The low resolution can be improved by using the constant Q transform (CQT) which is frequently used in auditory applications [17]. Unlike the STFT, the CQT provides a frequency resolution that depends on the geometrically spaced center frequencies of an analysis window [18]. In this paper, an adaptive method is proposed that provides an effective framework of switching between STFT for narrow band and CQT for wide-band signals, after analyzing the input signal. No prior information about the input signal is required in the proposed method. The proposed method is also capable of constructing a nonuniform filter bank according to user-defined parameters. This helps in the removal of filter bank redundancies. The results obtained from the proposed approach not only show an improved spectrogram visualization but also reduce the computation cost and show 87.71% of the appropriate window length selection.

## 2. Short Time Fourier Transform and Constant Q Transform

**Figure 1:**
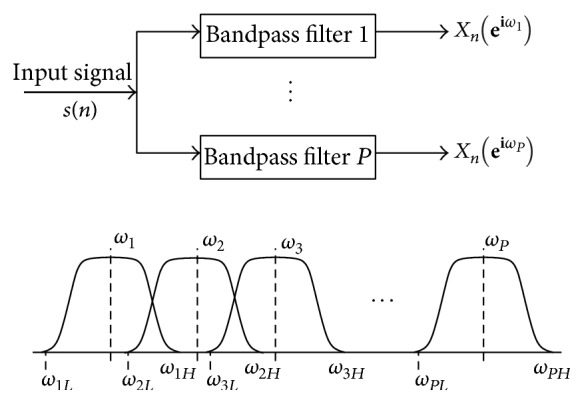
Uniform filter bank (STFT) with fixed time-frequency resolution.

**Figure 2:**
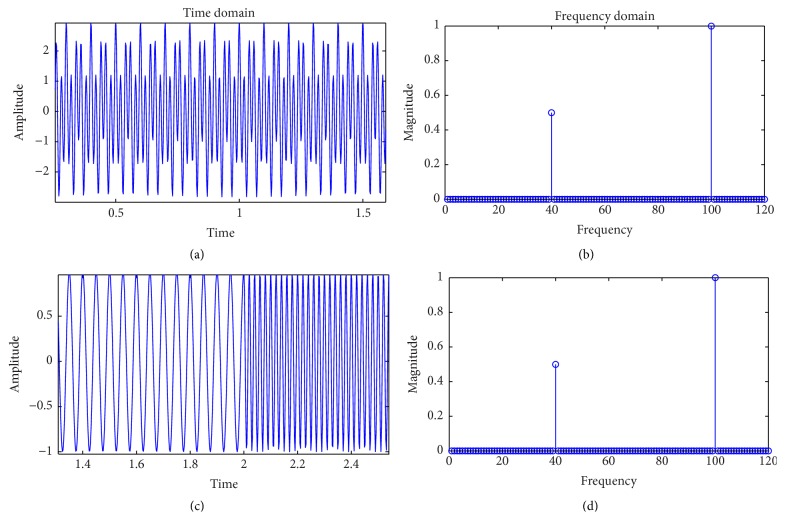
(a) Time domain representation of 40 Hz and 100 Hz combined signal for 2 seconds; (b) Fourier transform of part (a); (c) time domain representation of 40 Hz signal for first two seconds and 100 Hz signal for next two seconds; (d) Fourier transform of part (c).

**Figure 3:**
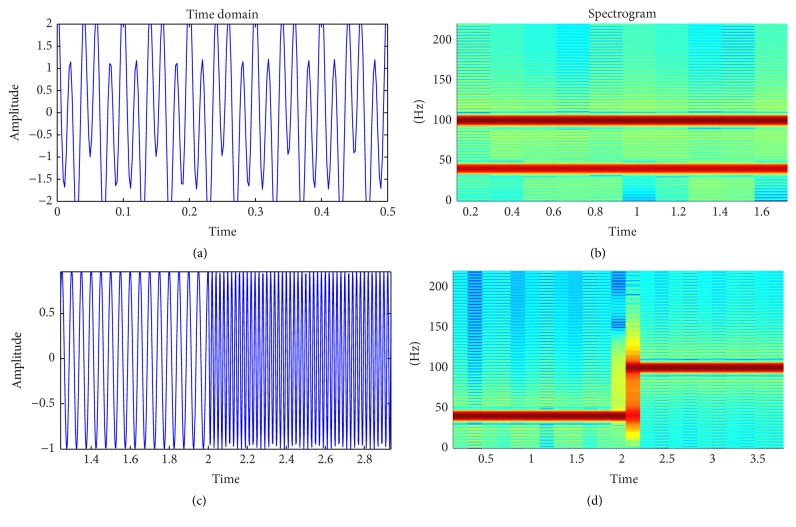
(a) Time domain representation of 40 Hz and 100 Hz combined signal for 2 seconds; (b) magnitude STFT representation of part (a); (c) time domain representation of 40 Hz signal for first two seconds and 100 Hz signal for next two seconds; (d) magnitude STFT representation of part (c).

The STFT is achieved by introducing a sliding window to the nonstationary signal. This window adds a new dimension of time to the frequency response. In the discrete time-case, this is represented as

(1) $$\begin{array}{c} X\left(\;n,\omega \;\right)=\overset{\infty }{\underset{m=-\infty }{\sum }}s\left(\;m\;\right)w\left(\;n-m\;\right)e^{-j\omega m}, \end{array}$$

where *n* and *k* are the time and frequency domain indices, *s* is the input signal, *w* is the window function, and *m* is the window interval centered around zero. The STFT can also be interpreted as a uniform filter bank [19]. The output signal *X*(*n*, *k*) is essentially the STFT (index *n*) obtained at the *k*th channel of the filter bank (Figure 1). The window function is assumed to be nonzero only in the window interval. As an example, (1) is applied to two signals. The first signal is a composite signal bearing frequencies of 40 Hz and 100 Hz. The second shows both the signals in isolation, occupying one-half of the time window each. As can be seen from the equivalent Fourier transform (Figure 2), the Fourier space cannot distinguish between the two types of signals. On the other hand, the distinction is clearly visible upon viewing the spectrogram of the STFT (Figure 3).

The time-frequency resolution of the spectrogram is dependent upon the chosen window size. A larger size will result in higher spectral, but lower temporal resolution, whereas the opposite will result in a lower spectral, but higher temporal resolution. This relationship is described as the* Uncertainty Principle* [20]. In this case, a variable window size would be ideal as it will provide high spectral resolution at low frequencies and high temporal resolution at high frequencies. A good candidate for achieving this is the constant Q transform (CQT) [21], where *Q* is the quality factor and its description appears shortly. Like the STFT, the CQT can also be interpreted as a filter bank. The only difference is that, in the case of CQT, the filters are geometrically spaced center frequencies such that the bandwidth Bw_*k*_ of the *k*th filter is a multiple of the (*k* − 1)th filter:

(2) $$\begin{array}{c} {Bw}_{k}=\left(\;2^{1/n}\;\right){Bw}_{k-1}, \end{array}$$

where *n* is the number of octaves per filter. As such, the bandwidth Bw_min_ of the lowest filter is given as

(3) $$\begin{array}{c} {Bw}_{k}={\left(\;2^{1/n}\;\right)}^{k}{Bw}_{min}. \end{array}$$

The quality factor *Q* is represented as the ratio of the center frequency *f*_*k*_ to the bandwidth:

(4) $$\begin{array}{c} Q=\frac{f_{k}}{{Bw}_{k}}. \end{array}$$

Due to variations, the window length for the *k*th filter is given as

(5) $$\begin{array}{c} N\left[\;k\;\right]=\frac{f_{s}}{{Bw}_{k}}. \end{array}$$

**Figure 4:**
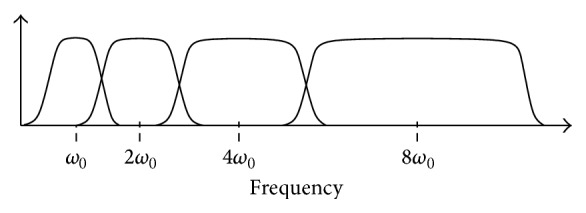
CQT filter bank with geometrically spaced window bins.

Finally, the CQT is given as

(6) $$\begin{array}{c} X_{CQ}\left[\;k\;\right]=\frac{1}{N\left[k\right]}\overset{N\left[k\right]-1}{\underset{n=0}{\sum }}W\left[\;n,k\;\right]x\left[\;n\;\right]e^{-j2\pi Qn/N\left[k\right]}, \end{array}$$

where *X*_*CQ*_[*k*] is the *k* component of the constant *Q* transform, *x*[*n*] is the input signal, and *w*[*n*, *k*] is the window function of length *N*[*k*]. The filter bank bearing geometrically spaced center frequencies of the CQT is shown in Figure 4.

## 3. Related Work

Time-frequency analysis methods are widely used in acoustics [22, 23], mechanics [5], electronics [24, 25], telecommunications [26, 27], biomedicine [28], and other fields involving processing of nonstationary information. Time-Frequency representation techniques are broadly categorized into parametric and nonparametric methods. Different parametric and nonparametric approaches have been studied in literature [29–35]. This paper deals with the nonparametric approach. An important and one of the most prevalent nonparametric tools is the STFT [1, 36] which has been discussed earlier in the introduction. The STFT is not desirable when dealing with wide and ultrawide-band signals which results in spectrogram resolution issues due to the size of the window [37, 38]. A number of techniques have addressed this issue. Spectrum analysis/synthesis can be added to the STFT as a feature [39]. Window size decisions can then be manually made on the basis of sinusoidal features of the signal such as peak amplitude, frequency, and phase trajectories. As such, two consecutive sinusoids with frequency difference Δ*f* can then be separated by setting the window size as

(7) $$\begin{array}{c} W=\frac{B_{s}F_{s}}{\Delta f}, \end{array}$$

where *W* is the window size (number of samples), *B*_*s*_ is the used window's main lobe size, and *F*_*s*_ is the sampling frequency. If no prior information is available regarding an input signal, then most of the existing methods follow the adaptive STFT that selects a window length from a pool of window sets [40–43]. This approach involves a high computation cost and the limited pool of window sets also reduces the chances of getting an accurate window length.

Various adaptively varying STFT approaches are proposed in [44] that reduce filter bank artefacts without compromising on time-frequency resolution. One of the approaches accounts for the time in which signal properties such as power and spectral shape remain preserved over the period, that is, a stationary region. Likewise, the opposite would be the time in which signal properties change over a period, that is, a transient region. Identifying a region involves integration of signal energy inside a given bank. The window size is then selected on the basis of variation of energies across critical banks. The general principle is increasing the time and frequency resolution for transient and nontransient regions, respectively. Similarly, a variable window length is determined by estimating the local instantaneous frequencies in every window slice over time in [45, 46].

Non-STFT based tools for time-frequency analysis also appear in the body of literature. Amongst these, the CQT [17, 47, 48] and the wavelet transform (WT) [49–52] are the most common. From the outset, both methods seem to be the same. However, the difference lies in the usage of the basis function. If the basis function can be interpreted as a windowed sinusoid, then both methods are essentially the same [53]. Wavelet transform can be categorized as discrete wavelet transform (DTW), continuous wavelet transform (CTW), and wavelet packet transform (WPT) [54]. The significance of wavelet transform depends upon the selection of appropriate wavelet basis because inappropriate wavelet basis will directly hamper the results of WT. Many publications have been seen, describing different wavelet basis and advancement in WT [55–60].

## 4. Proposed Method

**Figure 5:**
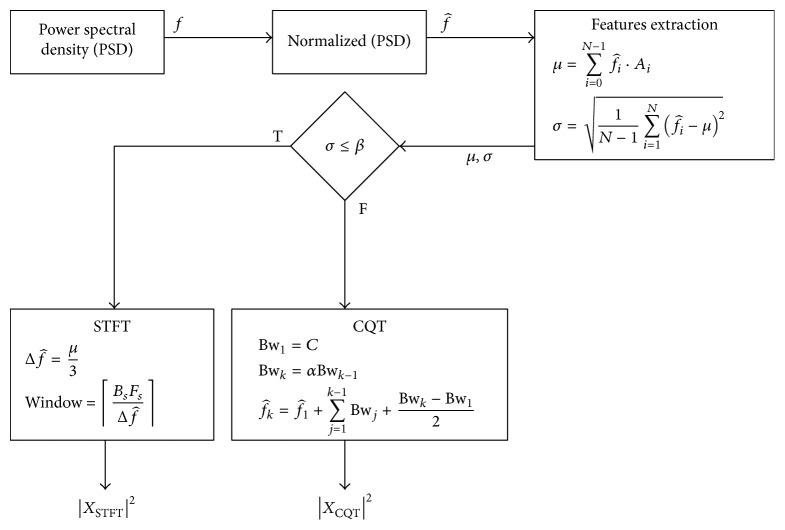
Block diagram of the proposed method.

Computationally, the CQT is expensive as compared to the STFT. The asymptotic complexity for the STFT is *O*(*n*log⁡*n*) following the pattern of the FFT, where *n* is the samples in the input signal. On the other hand, the asymptotic complexity of the CQT following (6) is *O*(*n*log⁡*n* + *nk* + *k*), where *k* is the number of components. For performance reasons, therefore, it would be better to select the STFT over CQT for visualization of the spectrum. However, the STFT is feasible only for narrow band signals where the filter bank with fixed window size is used. A simple but effective switching framework is proposed that can alternate between both tools after analyzing the input signal using spectrum sensing techniques. A block diagram of the proposed framework is shown in Figure 5.

The first step involves spectrum sensing that determines the orientation of the signal on the spectrum using the normalized power spectral density $\hat{f}$. The expectation *μ* and standard deviation *σ* is extracted from $\hat{f}$ as

(8) μ=∑iNf^i·Ai,

(9) σ=1N−1∑i=1Nf^i−μ2,

where *A*_*i*_ is the amplitude of normalized Power Spectral Density PSD ${\hat{f}}_{i}$. The expectation *μ* returns the frequency where PSD is concentrated. Together with *σ*, both give information about the distribution of the PSD. A signal would be considered narrow band when *σ* is smaller than a user-defined threshold *β*. An optimum threshold can be selected empirically such that smearing effect is minimized. After the analysis of known narrow and wide-band signals, the value of *β* is set to be 1500. The signals having *σ* less than 1500 are considered as narrow band signal and the appropriate tool; that is, STFT is selected. As mentioned earlier, STFT is computationally less expensive and the smearing effect is not prominent in case of narrow band signals. Signals having *σ* greater than 1500 are considered wide-band signal. In such scenario, the proposed method will adopt CQT tool. Unlike the STFT, CQT will minimize the smearing effect for wide-band signal and improve the visualization of spectrogram. The check will result in the selection of either the STFT or the CQT method as

(10) $$\begin{array}{c} \text{Tool}=\left\{\begin{array}{cc} STFT, & \sigma \leq \beta ,\\ CQT, & otherwise. \end{array}\right. \end{array}$$

Upon selection of STFT, the next step is to select an appropriate window size as [39], where two closest sinusoids can be distinguished using (7). However, nonstationary signals may involve a large number of sinusoids in close proximity. This results in a very small Δ*f* and consequently a large window. This makes the STFT very similar to the Fourier transform and will hamper temporal resolution. In order to select an appropriate window size a novel empirical model is proposed that adaptively selects a window size by modifying (7) to

(11) $$\begin{array}{c} W=\frac{3B_{s}F_{s}}{\mu }. \end{array}$$

Equation (11) will adopt an appropriate window size which does not lose any temporal information after the transform, where the size of the main lobe of the window *B*_*s*_ can be set to 2 for a rectangular, 4 for a Hamming/Hanning, and 6 for a Blackman window. In this work, Hamming window is used and the value of *B*_*s*_ is set as 4.

**Figure 6:**
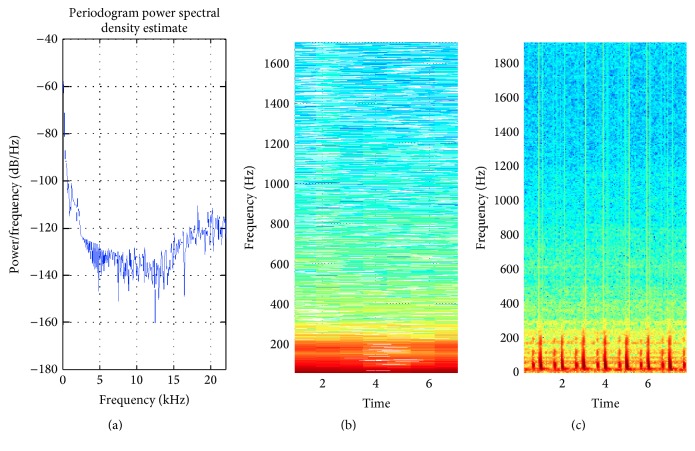
(a) PSD of heart signal; (b) STFT with default window; (c) STFT with proposed method window selection.

**Figure 7:**
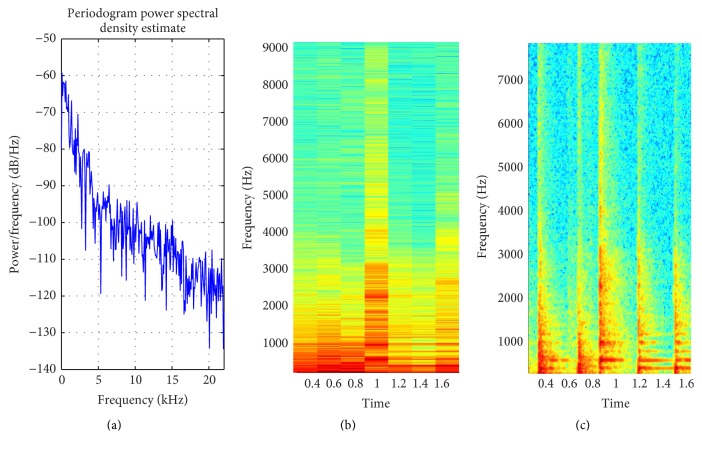
(a) PSD of mridangam signal; (b) STFT with default window; (c) STFT with proposed method window selection.

**Figure 8:**
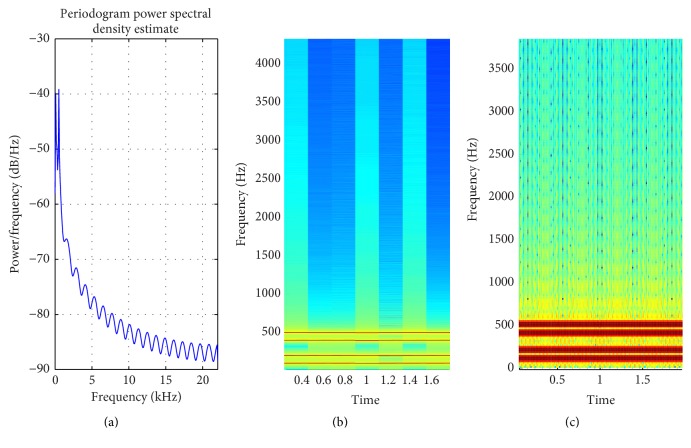
(a) PSD of multiple sinusoidals; (b) STFT with default window; (c) STFT with proposed method window.

**Figure 9:**
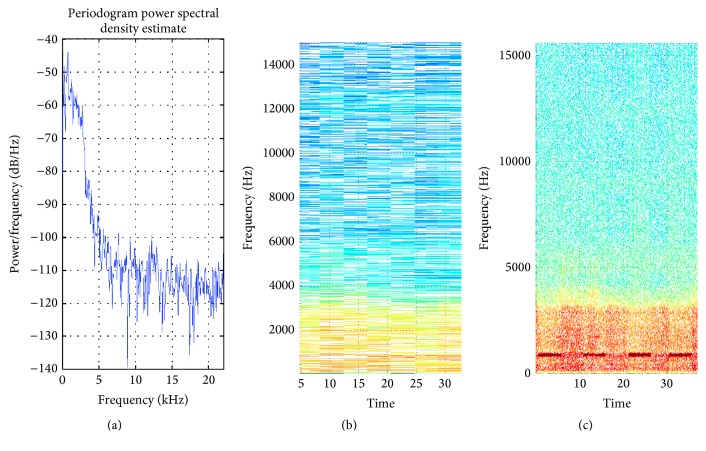
(a) PSD of radio signal; (b) STFT with default window; (c) STFT with proposed method window selection.

**Figure 10:**
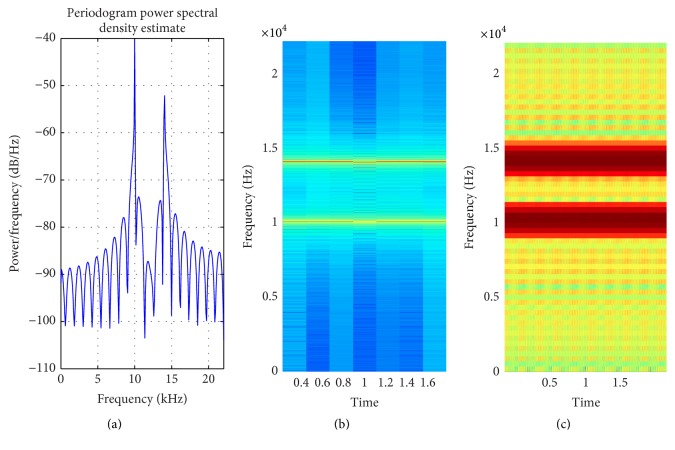
(a) PSD of high-carrier signals; (b) STFT with default window; (c) STFT with proposed method window selection.

**Figure 11:**
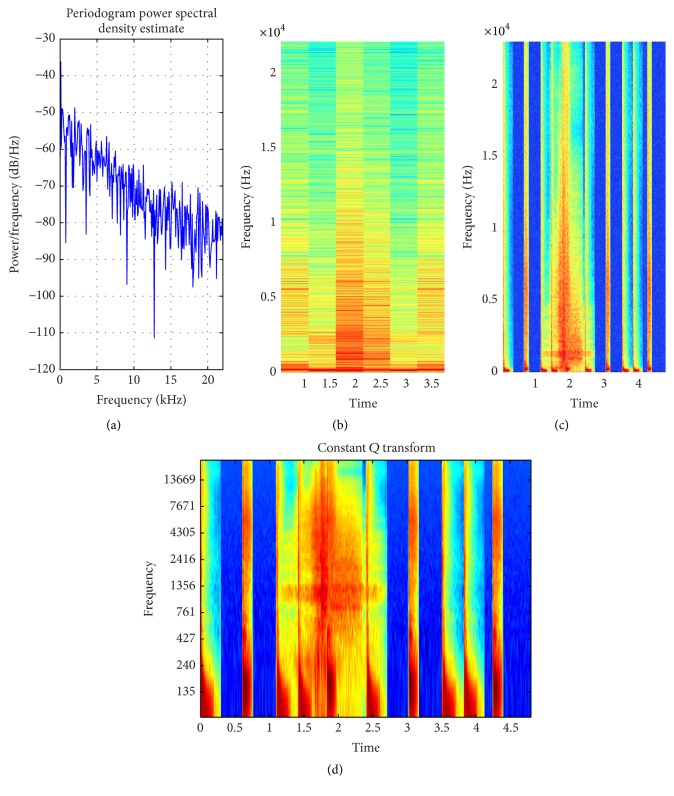
(a) PSD of music signal (wide band); (b) STFT with default window; (c) STFT with proposed method window selection; (d) magnitude of CQT (better time-frequency resolution achieved with CQT).

**Figure 12:**
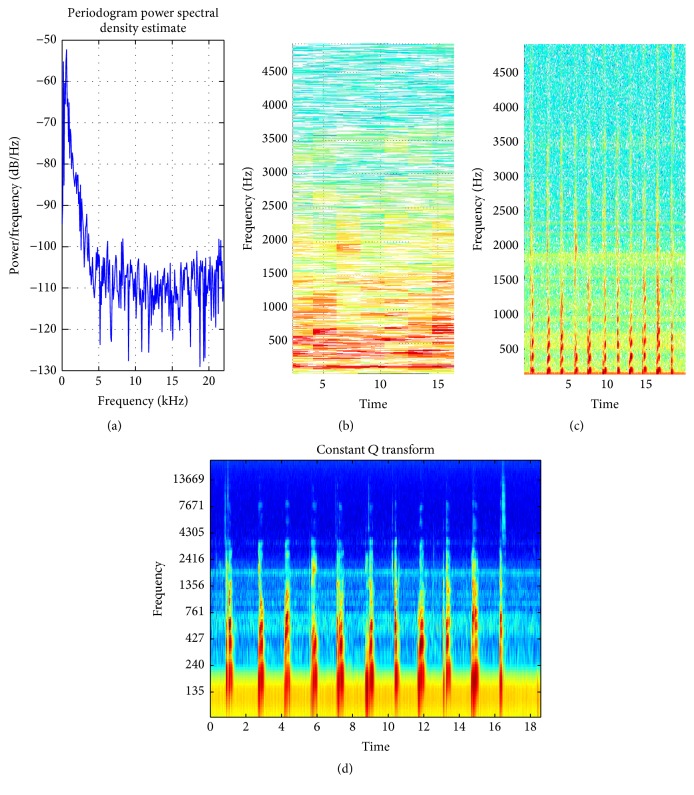
(a) PSD of speech signal (wide band); (b) STFT with default window; (c) STFT with proposed method window selection; (d) magnitude of CQT (better time-frequency resolution achieved with CQT).

The proposed method is tested over different inputs such as a heartbeat (Figure 6), mridangam (Figure 7), multiple sinusoids (Figure 8), radio (Figure 9), high-carrier (Figure 10), music (Figure 11), and a speech signal (Figure 12). According to the proposed method, five out of these seven signals are labeled as narrow band while the remaining two, music and speech, are labeled as wide-band signals. The proposed model adopts an appropriate window size for STFT using (11). All the figures show how the adaptive window selection improved the spectrogram visualization. The results from each signal type are given in Table 1.

A user-defined filter bank can be constructed using an approximation of the signal bandwidth (0.4–10 KHz) and its orientation using [61] as

**Figure 13:**
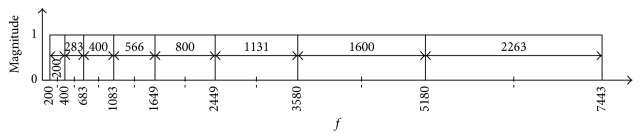
User-defined filter bank. Parameters provided by a user.

(12) Bwk=C,k=1,αBwk−1,2≤k≤Q,fk=f1+∑j=1k−1Bwj+Bwk−Bw12,

where *C* is the arbitrary bandwidth, *f*_1_ is the center frequency of the 1st filter, *α* is the logarithmic growth factor, and *Q* is the total number of filter banks. This will not only reduce the number of banks but will also cover the band where a signal may lie. An example of a filter bank is shown in Figure 13 bearing signal bandwidth of 7.2 KHz ([0.2,7.4] KHz), *C* = 0.2 KHz, *f*_1_ = 0.3 KHz, *α* = 1.4142, and *Q* = 8. The entire process of our proposed method is listed in Algorithm 1.

## 5. Results and Discussion

A quantitative analysis of the proposed method is discussed in this section. The method selects an appropriate window length *W* without prior information about the input signal. Considering a composite signal bearing frequencies 100, 200, 400, and 500 Hz, then the Hamming window length required to provide the frequency resolution of 100 Hz (Δ*f* = 200 Hz − 100 Hz) would be *B*_*s*_ *F*_*s*_/Δ*f* = 4 × 44100/100 = 1764.

This shows that the minimum window size required to get 100 Hz frequency resolution is 1764 samples [39]. By increasing the window size the frequency resolution increases but this will hamper the temporal resolution. The window length is set manually to 1764 samples in order to achieve the frequency resolution of 100 Hz. Background knowledge about the input signal is required to set the appropriate window length. The proposed method automatically calculates an appropriate window length using (11) as:

(13) Δf=μ3=386.133,W=BsFsΔf=1371.

Figure 8 shows how the proposed method adaptively selects the window size and improve the spectrogram. Signals that are almost invisible in default window size are explored by proposed method. The percentage of appropriate window length selection is 1371/1764 × 100 = 77.72%. In nature most of the signal are nonstationary and it is not possible to have information about all types of signal. Hence, it is very difficult to set an appropriate window length. The proposed method is evaluated on a number of nonstationary signals. Mridangam is an instrument which produces complex sound. The mridangam has got some stable harmonics and the minimum distance between two harmonics must be known in order to select an appropriate window length. After the analysis of mridangam signal, the first harmonic is around 200 Hz and the second harmonics is around 400 Hz. The minimum distance between two consecutive partials is around 200 Hz. So the appropriate window length is 882 samples. The adaptive window selected from the proposed method is 1003 samples. Hence, the percentage of appropriate window selection is 87.93%. Figure 7 shows that the proposed method improves the spectrogram by prominently displaying the harmonics which is not visible in default window selection. The proposed method is fully automatic and requires no prior information about the input signal. After the statistical analysis of input signal, the proposed method selects an appropriate window size using the empirical model proposed in this paper.

The heartbeat of normal human heart consists of *S*1 and *S*2 sounds. *S*1 results from mitral and tricuspid valve closure. It is a duller, lower-frequency sound than *S*2 and occurs at the beginning of ventricular systole. The approximate frequencies from different literatures for *S*1 and *S*2 are 20–120 Hz and 60–250 Hz, respectively. Hence, the appropriate window length to provide 30 Hz frequency resolution is 5880 samples. The window selected by the proposed method is 5816 samples. The percentage of appropriate window length is 98.91. Adaptive window clearly shows *S*1 and *S*2 signals which is completely missed in the default window as shown in Figure 6. A number of nonstationary signals are evaluated from proposed method, which is summarized in Table 2.

The appropriate window length is only possible when complete information about the input signal is known. This is usually not possible for all types of input signal. Hence, the proposed method is able to select an appropriate window size without any prior information about input signal and achieved the overall 87.71% of appropriate window length selection.

Note that the appropriate fixed window length is selected for narrow band signal. For wide-band signal it is not possible to select an appropriate fixed window length because long window length improves the spectral resolution at the cost of temporal resolution and vice versa. The proposed method is able to detect the wide-band signal and automatically selects constant Q transform that provides high spectral resolution at low frequency and high temporal resolution at high frequency with geometrically spaced center frequencies.

The existing methods for wide-band signal select window size from adaptive STFT using two main approaches. (1) Select a window size from a pool of windows using different concentration measurements such as skewness, kurtosis, and integrate energies [40–44]. (2) Define a benchmark *τ* and adjust it according to local characteristics of input signal using some concentration measurements such as instantaneous frequency and integrated energies [45, 46]. The problem with former approaches is that (i) they cannot obtain the optimal window length quickly or even fail to converge to the optimal window length and (ii) they are computationally expensive.

In [44] the smearing of energy in spectrogram is reduced by calculating STFT with 4 different window sizes. This increases the computational time approximately 3 times as compared to the proposed method. For all types of input signals whether narrow or wide-band signals, 4 different window sizes are used to reduce the smearing effect. The proposed method intelligently selects STFT for narrow band signal because for narrow band signal the fixed window length will not produce much smearing effects and improves the efficiency 4 times. When the input signal is wide-band signal then smearing effect is prominent while using STFT. In such a scenario, the proposed method selects CQT, which is computational expensive compared to STFT but it provides much better resolution and reduces the smearing effect. Figures 11(d) and 12(d) show the improved time-frequency resolution achieved by CQT.

The problem with the later approaches is that they are computationally expensive, which decides the window length on local characteristics of input signal. In [46] variable STFT is proposed, which adapts variable window length after analyzing the local characteristics of input signal. This is computationally expensive. The processing time for fixed STFT of length 64 and 128 is 0.1716 s and 0.1560 s, respectively, where the processing time of variable STFT is 0.5928 s for the same data. This demonstrates that the computing cost of variable STFT or any adaptive STFT which decides window length on local characteristics is much greater than the STFT. Variable STFT and adaptive STFT provide better resolution as compared to STFT but the proposed method solved the resolution problem by adapting CQT for wide-band signal. Hence, the proposed method not only is able to improve the time-frequency resolution but also reduces the computational cost. The computing costs are compared in Table 3.

## 6. Conclusion

In this paper, a general framework for effective multiresolution signal analysis has been demonstrated. The framework avoids the undesirable side effect of the STFT such as fixed time-frequency resolution for all types of input signals. After the analysis of input signal the method adapted an appropriate tool, that is, STFT and CQT for narrow and wide-band signal, respectively. The proposed method is capable of selecting an appropriate window length for STFT and achieved an overall of 87.71% of appropriate window length selection. The proposed method also allows a user to dynamically construct the filter bank according to the parameters provided by the user, which helps in the reduction of redundancy. The results obtained from the proposed method have improved spectrogram visualization and computing cost and achieved 87.71% of appropriate window length selection. The proposed method is fully automatic and required no prior information about the input signal. The results obtained from the proposed method directly contributes in different domains such as feature extraction, for example, harmonic, pitch, attack, delay, and energy. These features can be used in different applications such as speech and speaker recognition, biomedical signal analysis, and music instrument analysis. In future, the authors are planning to automatically build a desirable nonuniform filter bank after analyzing the characteristics of input signal. The filter bank will not be limited to linear or geometrical spacing only. The aim is to reduce the computing cost.

## Supplementary Material

The Proposed method is applied on various signals such as heart beat, speech, music (mridangam), radio, and carrier signals. The improved spectrogram of these signals obtained from our proposed method is shown in Figures 6–12. The supplementary materials of these signals are provided in S1–S7.

## Figures and Tables

**Algorithm 1:**
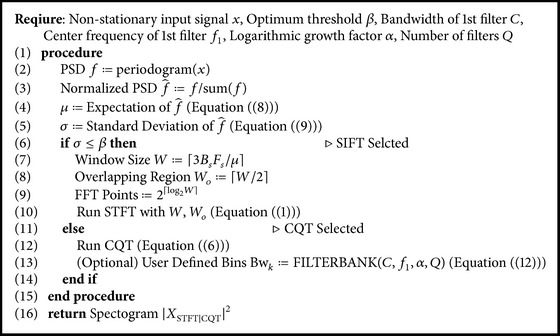
Complete algorithm.

**Table 1:** Adaptive window selection from proposed method, where *μ* is estimation, *σ* is the standard deviation, *β* is the optimal threshold (1500), and *W* is the window size.

Signal	Type	*μ*	*σ*	Decision	*W*
Heartbeat (Figure 6)	Low	90.99	135.49	STFT	5816
Mridangam (Figure 7)	Intermediate	527.61	706.89	STFT	1003
Carriers (Figure 8)	Intermediate	386.13	722.57	STFT	1371
Radio (Figure 9)	Intermediate	2632.8	542.37	STFT	201
High carrier (Figure 10)	High	10425	1117	STFT	51
Music (Figure 11)	Mixed	2170	2160	CQT	Variable
Speech (Figure 12)	Mixed	810.15	1302	CQT	Variable

**Table 2:** Adaptive window selection from proposed method, where *l*_*A*_ is the appropriate length and *l*_*P*_ is the proposed length.

Signal	Type	*l*_*A*_	*l*_*P*_	% achieved
Heartbeat (Figure 6)	Low	5880	5816	98.91
Mridangam (Figure 7)	Intermediate	882	1003	87.93
Carriers (Figure 8)	Intermediate	1764	1371	77.72
Radio (Figure 9)	Intermediate	176	201	87.56
Carrier (Figure 10)	High	44.1	51	86.47

**Table 3:** Adaptive short time fourier transform.

Schemes	CPU time (seconds)
STFT__fix=128__	0.1560
STFT__fix=64__	0.1716
CQT	0.413
VSTFT/ASTFT	0.5928
Proposed method	0.2845

STFT: Short Time Fourier Transform; CQT: constant Q transform; VSTFT: Variable Short Time Fourier Transform; ASTFT: Adoptive Short Time Fourier Transform.
